# Systematic Review of the Prognostic Role of the Immune System After Surgery of Colorectal Liver Metastases

**DOI:** 10.3389/fonc.2019.00148

**Published:** 2019-03-19

**Authors:** Joost Hof, Klaas Kok, Rolf H. Sijmons, Koert P. de Jong

**Affiliations:** ^1^Department of Hepato-Pancreato-Biliary Surgery and Liver Transplantation, University Medical Center Groningen, University of Groningen, Groningen, Netherlands; ^2^Department of Genetics, University Medical Center Groningen, University of Groningen, Groningen, Netherlands

**Keywords:** colorectal liver metastases, immune system, prognosis, survival, immunohistochemistry, high-throughput

## Abstract

**Background:** The current prognostication of patient survival after surgery for colorectal liver metastases is based on clinical characteristics, but low accuracy makes it difficult to guide treatment for the individual patient. Rapidly evolving technologies have led to the expectation that biomarkers will be able to outperform the current clinical scoring systems and provide more effective personalised treatment. Two main topics prevail in cancer treatment, namely the role of the immune system and the prediction and prognostication by application of high-throughput methodology. The aim of this review is to examine the evidence for prognostic immunological and molecular markers studied in tumour tissue obtained at surgical resection for colorectal liver metastases.

**Methods:** First we analysed immunophenotypical protein markers, that are mainly studied by immunohistochemistry. Second, we review molecular markers by analysing high-throughput studies on tumour mRNA and microRNA expression.

**Results:** CD3, CD4, and CD8 are the most frequently studied protein markers. High intra-tumoural CD3+ T cell infiltration and low CXCR4 expression have the best association with favourable patient survival. Studies that analysed microRNA or mRNA expression data showed very little overlap in prognostic genes.

**Conclusions:** Patient prognostication after surgery for colorectal liver metastases by analysing the immune system remains difficult. Current data are based on diverse and heterogeneous patient populations which prohibits drawing firm conclusions.

## Introduction

### Rationale

In Europe, colorectal carcinoma (CRC) is the cancer with the third highest incidence and the second highest mortality rate (1). The liver is the most common site of metastases, and a curative resection of colorectal liver metastases (CRLM) is impossible in 75–80% of patients because of widespread liver involvement, extra-hepatic disease or comorbidity (2). Although recent advances in the treatment of CRLM have extended the possibilities to increase curability, disease will still recur in many patients undergoing a potentially curative resection (3). The mean 5-year survival of patients undergoing intentionally curative surgery varies from 15 to 60% (4).

Models that predict the outcome of treatment in patients with CRLM can support the therapeutical management of individual patients. To this end, several prognostic scoring systems have been developed to guide treatment decisions, predominantly based on clinicopathological characteristics. Unfortunately, these clinical models still have high variability and a review of these models found no common prognostic factor between them (5). Multiple prognostic factors have been identified in patients with CRLM, e.g., KRAS and BRAF mutational status and surgical resection margin (5–7). Rapidly evolving technologies have led to the expectation that immunological and molecular biomarkers will be able to outperform the current clinical scoring systems and provide more effective personalised treatment.

### Objectives and Research Question

Two main topics prevail in cancer treatment, namely the role of the immune system (8) and the prediction and prognostication by application of high-throughput methodology. The latter has been shown to be of great prognostic value in for instance breast cancer (9). Both topics are not recently analysed in a systematic review on the treatment of CRLM. Therefore, the aim of this review is to examine the evidence for prognostic immunological and molecular markers studied in tumour tissue obtained at surgical resection for CRLM. To do this, we first review studies using tissue-based immunophenotypical protein markers. We then review tissue-based molecular markers by assessing tumour mRNA and microRNA expression, focusing on genes related to the immune system.

## Methods

### Study Design and Search Strategy

This systematic review is based on literature that analyses the effectiveness of tissue-based prognostic markers of patient survival and recurrence rate after surgery for CRLM. Our review examines papers published in English between January 1, 2005 until November 10, 2017. We chose 2005 as the starting year because the last systematic review on prognostic markers included papers until 2005 (10). Online publications ahead of print were also included. The databases of Pubmed and Web of Science were screened using the search terms: “colorectal liver metastases” OR “colorectal liver metastasis” AND “tumour biology” OR “tumour biology” OR “genetic” OR “genetics” OR “molecular” OR “markers” OR “expression” OR “mutation” OR “mirna” or “microRNA” OR “lncrna” OR “DNA” OR “RNA.” We first screened the studies for eligibility based on title and abstract, and then thereafter on reading the entire manuscript. References cited in the studies were also checked to identify other relevant papers not found in our initial search. After this initial search process, we also performed searches on all the individual immunological markers, e.g., using search terms like: “CD4” AND “colorectal liver metastases.” In all instances, the data used in this review was extracted from the original papers.

### Data Collection and Analysis

The following variables were collected: immune markers, patient survival, administration of chemotherapy, scientific method, and statistics. Survival rates in the various studies were variable and were recorded as median survival (in months), overall survival (OS), cancer-specific survival (CSS) or disease-free survival (DFS). Additionally, disease specific survival (DSS) was recorded as CSS, and recurrence-free survival and progression-free survival were recorded as DFS. To assess the risk of bias in the selected studies, several study and patient characteristics were analysed, including administration of neoadjuvant and adjuvant chemotherapy, number of patients and the use of univariable/multivariable analyses. However, reporting bias in the original papers could not always be assessed, with non-significant findings in particular not always reported. The principal summary measures are the correlation of the immune-related markers with patient survival. The data was analysed by combining the results of the selected studies.

## Results

### Study Selection

**Figure 1:**
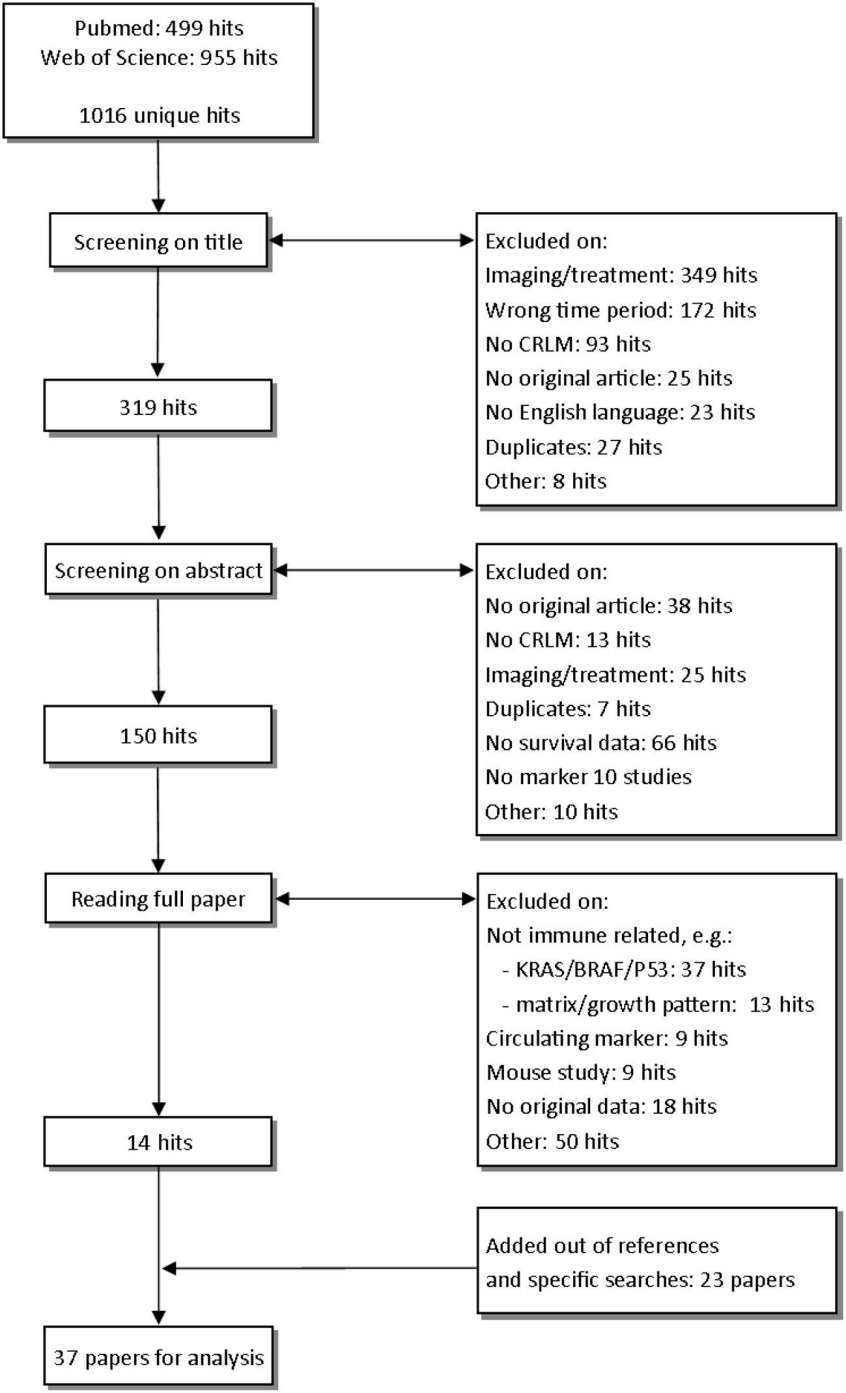
Selection of studies.

The studies examined in this review are shown in Figure 1. In total, after removing duplicates, we identified 1,016 unique hits using our search terms. After screening on title and abstract, we read 150 studies in full. After reading the full paper, 14 papers were eligible for analysis. In cited references and specific searches, we found another 23 eligible studies, resulting in the 37 studies that we discuss in this review (Figure 1).

### Immunophenotypical Protein Markers

Phenotypes of tumour infiltrating inflammatory cells and profiles of cytokines were mostly studied by immunohistochemistry (IHC) on tissue sections, by scoring the presence of tumour infiltrating lymphocytes (TILs) in the intra-tumoural and peri-tumoural region. In contrast, studies that use tissue microarrays (TMA) for the IHC studies only analyse intra-tumoural areas. Distant liver parenchyma is seldom studied. The largest proportion of TILs in the tumour microenvironment consists of T cells, which are further subdivided according to their IHC staining pattern. The most commonly used markers are CD3 (all T cells), CD4 (T helper cells), CD8 (cytotoxic T cells), and FOXP3 (regulatory T cells). Supplementary Table 1 shows the summary of all studies that analyse immune-related protein and RNA expression.

#### General T Cells

In eight studies, CD3+ was used as a marker for T cells. All eight analysed intra-tumoural expression and five also analysed peri-tumoural regions (Table 1). An association between high infiltration of intra-tumoural CD3+ T cells and favourable patient survival was seen in 5/8 studies (11–15) analysing a total of 444 patients, while the remaining three studies (16–18), analysing 426 patients, yielded non-significant findings. The difference in prognostic value of CD3+ seems to be associated with neoadjuvant chemotherapy. That is, in the studies that show an association between high intra-tumoural CD3+ T cells and favourable patient survival, more patients received neoadjuvant chemotherapy (176/235; 74.9%) (13, 15) compared to studies that show no association with patient survival (85/270; 31.5%) (16, 17). Five studies analysed the peri-tumoural regions (12–15, 17), three showed no association with patient survival (13–15), one showed a favourable DFS (17), and one showed an unfavourable OS (12). In this latter paper, only 3 out of 36 patients had tumours with high numbers of CD3+ cells, yielding results that are difficult to interpret (12). In addition, one study analysed general T cells by quantification of CD45 and showed an association between a high peri-tumoural infiltration and a favourable survival (19).

**Table 1:** Studies that analyse CD3+, CD4+, and CD8+ T cells by IHC and flow cytometry.

**Author**	**Method**	**N**	**Neo**	**Adj**	**Location**	**High CD3 yields**	**Statistics**	**High CD4 yields**	**Statistics**	**High CD8 yields**	**Statistics**
Brunner et al. (20)	IHC	201	90^*^	NR	intra-tumoural			not sign.	UV (*p =* 0.317)	not sign.	UV (*p =* 0.713)
					peri-tumoural^a^			better OS	UV (*p =* 0.003)	better OS	UV (*p =* 0.002)
Katz et al. (16)	IHC TMA	188	47^*^	166	intra-tumoural	not sign.	UV (*p* = 0.36)	better OS and DFS	MV (*p =* 0.02 & *p =* 0.04)	better OS	UV (*p =* 0.04) MV (*p =* 0.08)
Nakagawa et al. (21)	IHC	162	65^*^	NR	intra-tumoural			not sign.	UV (*p =* 0.11)	not sign.	UV (*p =* 0.48)
					peri-tumoural			not sign.	UV (*p =* 0.06)	not sign.	UV (*p =* 0.10)
Katz et al. (11)	IHC TMA	162	NR	NR	intra-tumoural	better DSS	MV (*p =* 0.04)	worse CSS	MV (*p* < 0.001)	better CSS	MV (*p* < 0.001)
Cavnar et al. (18)	IHC TMA	156	NR	NR	intra-tumoural	not sign.	UV (*p =* 0.9)	better OS and DFS	UV (*p* = 0.04 and *p =* 0.025)	not sign.	UV (*p =* 0.32)
Donadon et al. (13)	IHC	121	96^*^	64	intra-tumoural	better OS	MV (*p* = 0.005)				
					peri-tumoural	not sign.	UV (*p =* 0.458)				
Mlecnik et al. (15)	IHC	114	80^*^	77	intra-tumoural	better OS	UV (*p =* 0.009)			Better OS and DFS	UV (*p =* < 0.001 and *p =* 0.004)
					peri-tumoural	not sign.	UV (*p =* 0.23)			Better OS and DFS	UV (*p =* 0.02 and *p =* 0.027)
Tanis et al. (17)	IHC	82	38^*^	38	intra-tumoural	not sign.	UV (NR)	not sign.	UV (NR)	not sign.	UV (NR)
					peri-tumoural	better survival	UV (*p =* 0.031)	not sign.	UV (NR)	not sign.	UV (NR)
Berthel et al. (12)	IHC	36	NR	NR	intra-tumoural	better OS	UV (*p =* 0.05)			better OS	UV (*p =* 0.05)
					peri-tumoural	worse OS	UV (*p =* 0.05)			not sign.	UV (NR)
Pugh et al. (14)	Flow cy-	11	NR	NR	intra-tumoural	better OS	UV (*p =* 0.018)				
	tometry				peri-tumoural^b^	not sign.	UV (*p* > 0.1)				

*N, number of patients; Neo, neoadjuvant chemotherapy administered; Adj, adjuvant chemotherapy administered; NR, not reported; UV, univariable analysis; MV, multivariable analysis*.

* *a certain percentage of patients in this study were administered to oxaliplatin-based neoadjuvant chemotherapeutic regimens*.

a *“near stroma” was noted down as peri-tumoural*.

b *“peri-tumoural liver” was noted down as peri-tumoural*.

#### T Helper Cells (CD4) and Cytotoxic T Cells (CD8)

Out of the eight papers that reported on CD4+ and CD8+ T cells, six reported on both markers (11, 16–18, 20, 21) and two only on CD8 (12, 15) (Table 1). Interestingly, out of the six papers that studied both CD4 and CD8, four described similar associations with patient survival related to these two markers (16, 17, 20, 21) (Table 1). An association between high intra-tumoural infiltration of CD4+ T cells and favourable patient survival was described in 2/6 papers (16, 18) analysing a total of 344 patients, while three other studies analysing 544 patients yielded non-significant results (17, 20, 21). In contrast, one study showed an association between a high intra-tumoural CD4 expression and unfavourable patient survival (11). Additionally, in the papers that study peri-tumoural regions (17, 20, 21), an association between high peri-tumoural infiltration of CD4+ T cells and favourable patient survival was seen in 1/3 papers (20). Four out of eight papers describe an association between high intra-tumoural CD8+ T cells and a favourable patient survival (11, 12, 15, 16), analysing a total of 500 patients, while the other 4 studies, analysing 601 patients, observed no such association (17, 18, 20, 21). In addition, two out of five studies describe an association between high CD8+ T cells in the peri-tumoural regions and favourable patient survival (15, 20), while this association was not observed in the other three studies (12, 17, 21) (Table 1). Of note, one study analysed granzyme B positive immune cells by IHC, which is primarily a marker for cytotoxic T cells, and did not find an association with patient survival (12). Based on these papers it is tempting to suggest that high intra-tumoural infiltration of CD4+ and CD8+ T cells might be associated with a favourable survival, but it should be realised that this was observed in the minority of studies (CD4: 2/6 studies; CD8: 4/8 studies). In the peri-tumoural regions, an association with patient survival has not been observed. The assessment of bias by chemotherapeutical treatment is limited, as not all studies report the use of chemotherapy (Table 1).

#### FoxP3+ Regulatory T Cells (Tregs)

Tregs are known for their immunosuppressive function in tumour biology. Of the four studies that fulfilled criteria for analysis in this review, three did not show an association between intra-tumoural FoxP3+ T cells and patient survival (16, 18, 21). One other group also studied intra-tumoural FoxP3+ T cells, but did not report on a survival analysis in relation to this marker, strongly suggesting that there is no association with patient survival (15). Contradictory results have been reported for FoxP3+ T cells in peri-tumoural regions. While one group showed an association between high FoxP3+ T cells and a favourable DFS [26], this was not observed in another study (15).

#### Memory T cells

Memory T cells are T cells that encounter and respond to an antigen, and are therefore primed to react much faster to a second encounter with that antigen (22). The marker CD45RO is used to detect memory T cells. Three independent studies did not show an association between intra-tumoural presence of CD45RO+ T cells and patient survival (11, 15, 20). Two of these studies also analysed the peri-tumoural regions (15, 20). In one study analysing 201 patients by IHC, high infiltration of CD45RO+ T cells near the tumour border was associated with a favourable patient survival (20).

#### CD20+ B cells

B cells are lymphocytes that can serve as antigen presenting cells, and they are known for secreting antibodies and cytokines. The association of CD20+ B cells with patient survival was analysed in four independent studies. Two studies showed an association between high peri-tumoural CD20+ B cells and a favourable overall survival (15, 19). In addition, one of these studies also showed an association between high intra-tumoural CD20+ B cells and a favourable OS and DFS (15). The two other studies observed no associations with patient survival (12, 17).

#### Macrophages

The innate immune system is the first line of defence against pathogens, with macrophages as the most-studied immune cell type in oncology, which is mainly stained using CD68 or CD163. In one study, the presence of more intra-tumoural CD68+ cells was associated with a favourable patient DFS (18). Five other studies reported no association between the number of macrophages and patient survival (12, 17, 19, 23, 24). In three out of these five studies, CD68+ (17, 24) and CD163+ (12) macrophages are not mentioned in the survival analysis, strongly suggesting that there is no significant association. Another study specifically analysing the peri-tumoural regions used MAC387 as macrophage marker and found no association with patient survival (23). MAC387 is thought to represent blood-derived macrophages and monocytes recruited to the inflammatory site (25). In conclusion, although there are six studies analysing macrophages in the tumour microenvironment, only one study found an association between favourable survival and high CD68+ macrophages.

#### Natural Killer (NK) cells

NK cells are a type of cytotoxic lymphocytes that belong to the innate immune system, and they have been linked to antitumour activity in primary CRC (26). One flow cytometry-based study showed no association between patient survival and infiltration of NK cells (CD56+CD3–) (14). In contrast, another study showed that the presence of intra-tumoural NKp46+ cells was associated with a favourable OS after multivariable analysis. They also showed that peri-tumoural NKp46+ cells were not associated with patient survival, suggesting that the NK cells act intra-tumoural (13).

#### Mast Cells

Although mast cells are mainly known for their IgE response to allergies, they could also be involved in primary CRC biology (27). One study showed that high infiltration of CD117+ mast cells in CRLM tumour tissue is associated with a favourable DFS (17), while another study showed that high infiltration of peri-tumoural tryptase+ mast cells is associated with an unfavourable OS (23). In other cancers, high tumour-infiltrating tryptase+ mast cells generally have an unfavourable association with survival (28). The differences in markers and localisation precludes any conclusion on the role of mast cells in CRLM.

### Combining Multiple Immunophenotypical Protein Markers

In primary colorectal cancer a semiquantitative scoring system has been proposed, the Immunoscore, that quantifies CD3+ and CD8+ expression both intra-tumoural and peri-tumoural (29). Moreover, several studies developed their own IHC-based immunological score because it delivered the best explanation for the differences in patient survival they observed in their dataset. Three studies in CRLM showed significant associations between a high immunoscore and favourable patient survival, two in a univariable analysis (17, 30) and one in a multivariable analysis (15). Turcotte et al. showed that the combination of high CD3+ T cells and high MHC class I expression was the best predictor of long term survival (31). Another study showed that a high intra-tumoural expression of both CD3+ T cells and NKp46+ NK cells was associated with a favourable OS (13).

Katz et al. found that the combination of intra-tumoural CD4+ and CD8+ quantification was the best predictor of patient survival (11). In a more recent study, the same authors showed that a high intra-tumoural CD8+/CD3+ ratio was associated with a favourable OS, while a high FoxP3+/CD4+ ratio and a high FoxP3+/CD8+ ratio were independently associated with an unfavourable survival (16). Another study showed an association between a favourable patient survival and a high ratio of CD45RO+ cells concerning localisation (high peri-tumoural CD45RO+ and low CD45RO+ in distant liver parenchyma). In addition, they combine the CD45RO ratio with the histopathological growth pattern to create their best predictor of patient survival (20). Of note, a higher number of peri-tumoural TILs has been reported to be associated with the desmoplastic growth pattern of CRLM, which may explain the relative favourable prognosis compared to the replacement or pushing growth types (32). Finally, Mlecnik et al. show that the combination of high CD8 and CD20 expression, in both the intra-tumoural and peri-tumoural region, was associated with a favourable OS and DFS (15).

### Inflammatory Mediators

Cytokines and other inflammatory mediators can attract immune cells to the site of inflammation or have a promoting or repressing effect on the immune system. For example, CTLA-4 and PD-L1 are inhibitory ligands that can de-activate a proper T cell response toward the tumour and are the two primary targets of modern-day immunotherapy. One study in CRLM showed an association between a high PD-L1 expression in the tumour stroma and a favourable DFS (33).

#### CXC Chemokines and Receptors

CXCR2 is a chemokine receptor that mainly attracts neutrophils and endothelial cells. In addition, CXCL7 is an agonist of CXCR2 and is a cleavage product that is particularly released by platelets. In tumourigenesis however, CXCR2 might facilitate tumour growth and development of metastases by increased angiogenesis (34). Desurmont et al. showed that both a high CXCR2 and CXCL7 expression have an independent association with an unfavourable patient survival (35). Several other chemokines have been studied, but did not show an association with patient survival [e.g., CXCR7/CXCL12 (33), CXCL8 (34)].

#### CXC Chemokine Receptor 4 (CXCR4)

CXCR4 executes its effect by binding to its ligand CXCL12 (SDF-1), allowing downstream signalling that can alter several biological pathways including immune checkpoints (36). It has been shown that an inhibition of CXCR4/CXCL12 can improve the efficacy of immunotherapy, suggesting that high CXCR4 expression inhibits an effective immune response by targeting CTLA-4 (37). Out of the six papers that study CXCR4, four show an association between high CXCR4 expression and unfavourable patient survival (Table 2) (33, 38–40). The two other studies did not find significant associations with patient survival (35, 41). There is considerable variation in the methods used to quantify protein expression by IHC, particularly in the CXCR4 studies. Studies use different cut-off values for assessing positive staining [10% (40) vs. 50% (33)]. Another paper assessed CXCR4 expression using a digital slide computer scanner to score stromal or cancer cells for intensity and frequency (41). Finally, a study classified cytoplasmic CXCR4 expression as low or high relative to the staining intensity of hepatocytes (39). These different scoring methods and cut-off levels make it difficult to draw valid conclusions. However, CXCR4 is associated with an unfavourable prognosis in different scoring systems.

**Table 2:** Studies that analyse CXCR4 expression by IHC and/or qPCR.

**Author**	**N**	**Neo**	**Adj**	**Method**	**High intra-tumour al CXCR4 expression**	**Statistics**
Yopp et al. (40)	75	NR	10	IHC	unfavorable DFS	MV (*p =* 0.006)
D'Alterio et al. (33)	33	33	33	IHC [qPCR not sign. (*p =* NR)]	unfavorable DFS and CSS	MV (*p =* 0.004 and *p* < 0.001)
Kim et al. (38)	29	0	29	qPCR (IHC NR)	unfavorable OS	MV (*p =* 0.046)
Sakai et al. (39)	92	NR	NR	IHC	unfavorable OS	MV (*p =* 0.027)
Goos et al. (41)	507	60	97	IHC TMA	not sign.	UV (*p =* 0.33)
Desurmont et al. (35)	55	31	0	qPCR	not sign.	NR

*N, number of patients; Neo, neoadjuvant chemotherapy administered; Adj, adjuvant chemotherapy administered; NR, not reported; UV, univariable analysis; MV, multivariable analysis*.

#### Monocyte Chemoattractant Protein-1 (MCP-1)/CCL2

MCP-1 is a chemokine that mainly influences monocytes and macrophages; it can recruit macrophages via chemotaxis to promote tumour progression (42). One study showed that high MCP-1 expression was associated with unfavourable patient survival and increased hepatic recurrences after surgery. However, a significant correlation between MCP-1 and CD68+ macrophages was not found (24).

#### COX-2/PTGS2

COX-2 is thought to modulate prostaglandin pathways into promoting tumour immune evasion, and could therefore be a potential target for treatment (43). One group showed that a high intra-tumoural COX-2 expression measured by IHC was associated with an unfavourable OS in multivariable analysis (41, 44).

### Molecular RNA Expression Markers

Studies that analyse mRNA or miRNA expression generally only assess intra-tumoural expression of molecular markers. All expression studies that analyse molecular markers initially perform a high-throughput method to scout for interesting genes. In this review, we analysed the overlap between the high-throughput studies to assess if the immune system has a prominent role in identifying differences in patient survival.

#### Messenger RNA Expression

Table 3 shows the results of five papers that describe gene signatures associated with patient survival based on high-throughput expression microarrays (45–49). Balachandran et al. (48) used the test-set from Snoeren et al. (46) and the validation set from Ito et al. (45), thereby creating a validated multi-centre gene signature. Second, Snoeren et al. published two expression studies, with the second zooming in on stage II/III CRC patients (49). Interestingly, the four presented individual centre gene signatures [excluding Balachandran et al. (48)] do not share any gene and do not point toward a specific biological pathway. Of note, because these high-throughput studies do not show the total list of all genes and their association with survival, there could be overlap between the studies. We analysed the enrichment of the immune system in these five gene signatures by selecting the 76 genes and correlated this with the “immune system process” pathway in the Gene Ontology (GO) database (50, 51). Ten signature genes showed overlap with all immune related genes (*BNIP3, C1ORF218, CDC2L5, COLEC11, ITGB5, PLA2G2A, RNF135, RPS24, SERPINB1, ULBP2*). By specific searches, we found three additional genes related to the immune system (*BAT2, REG4, RIPK4)*. In total, 13 out of 76 (17.1%) genes were present in immune related pathways (marked bold in Table 3), which is comparable to all known immune related genes divided by all human genes (3119/20226; 15.4%) (50, 51). In conclusion, the presented gene signatures based on high-throughput expression profiling generate non-overlapping gene signatures and do not show predominant immunological characteristics that are associated with patient survival.

**Table 3:** High-throughput studies of mRNA expression.

**Studies**	**Ito et al. (45)**	**Snoeren et al. (46)**	**Snoeren et al. (49)**	**Balachandran et al. (48)**	**Van der Stok et al. (47)**
Nr of patients	96	119	30	96 test [Ito et al. (45)]	63
			(only CRC stage II/III)	119 validation [Snoeren et al. (42)]	
Experimental methods	Illumina, HumanHT-12 Gene Chip v3.0 Expression Beadchip	Qiagen, Human Array-Ready Oligo set (version 2.0)	Qiagen, Human Array-Ready Oligo set (version 2.0)	See Ito et al. (45) and Snoeren (46)	Illumina, HumanHT-12 v4 Expression BeadChip
Statistics	Supervised principle component method	Multivariate cox regression	MAANOVA	Multivariate cox regression	Leave one out cross validation
Neoadjuvant chemo	72%	In DFS ≤ 1 year: 62.5%	In DFS < 6 months: 29.4%	See Ito et al. (45) and Snoeren (46)	In DFS ≤ 1 year: 0%
		In DFS > 1 year: 40.4%	In DFS > 24 months: 15.4%		In DFS > 3 year: 0%
Adjuvant chemo	82%	In DFS ≤ 1 year: 45.8%	In DFS < 6 months: 94.1%	See Ito et al. (45) and Snoeren (46)	In DFS ≤ 1 year: 0%
		In DFS > 1 year: 74.5%	In DFS > 24 months: 53.8%		In DFS > 3 year: 0%
Genes present in	BAG3	***BAT2***	AMPD1	CES2	CASS4
signature	**C1ORF218**	C6orf141	ARL6IP5	DKK1	CLRN3
	C1ORF71	CCDC85A	ASAP2	DNAJC12	COX6A1
	***CDC2L5***	CPLX1	***BNIP3***	FGFBP1	ERN1
	CHN2	FAM174B	C13orf3	HOXC6	G3BP2
	CKS2	FRMD6	***COLEC11***	LRP8	***ITGB5***
	FGFBP1	GPR143	FYTTD1	LRRC42	JARID1A
	FLJ39632	hsa-mir-103-2	GDF15	NUP62CL	KIAA0319
	HSGT1	ITSN1	GTF3C3	ODC1	RAD9A
	LRRC42	KIAA0562	LAPTM4A	***PLA2G2A***	RPUSD1
	PCBD1	MAPKAPK2	LYPLAL1	PLCB4	***ULBP2***
	PLCB4	MYNN	SERPINB5	RAD23B	
	RAD23B	OR5P2	SMYD2	RBBP8	
	***RNF135***	OTUD5	THEM2	***REG4***	
	***RPS24***	PARN		***RNF135***	
	SLC28A3	***RIPK4***		RPS24	
	TIMM23	RP11-347C12.2		***SERPINB1***	
	ZNF827	ZNF134		SMIM24	
		chrX:142692034-142692102		STEAP1 TS	
					

*Five studies report on high-throughput mRNA expression. We report on the number of patients, the experimental method and protocol, and the statistical method. Furthermore, we report the percentage of patients in the different experimental patient groups that received neoadjuvant and/or adjuvant chemotherapy. Finally, we show the presented gene signatures that predict patient survival in these four different studies. Genes related to the immune system are in bold and italics*.

#### LIGHT/TNFSF14

Based on expression microarray results (45), it was validated by IHC that both a high LIGHT expression in the tumour cells and in TILs was associated with improved patient survival (52). Tumour necrosis factor superfamily member 14, also known as LIGHT, has been shown to induce T cell proliferation and tumour regression *in vivo* (53).

#### MicroRNA Expression

MicroRNAs are small non-coding 18-22nt RNAs that function in the post-transcriptional regulation of gene expression, primarily by silencing mRNA (54). Table 4 shows studies included in this review reporting on microRNAs and patient survival (55–62). There are eight high-throughput studies, of which seven validated their observations by qPCR (55–57, 59–62). In addition, in the earlier described expression study of Snoeren et al. (46), a high microRNA 103-2 expression was associated with an unfavourable DFS (Table 3). There is little overlap between the microRNA studies but, remarkably, the study of Ellermeier et al. shows opposing results compared to other studies (Table 4) (58). For example, Li et al. (59) observed an association between a high intra-tumoural microRNA 99b-5p expression and favourable patient survival, yet Ellermeier et al. show an association with unfavourable patient survival (58). Both studies differ in patient numbers and methodology (Table 4; qPCR array vs. microarrays). In conclusion, there is no overlap in differentially expressed microRNAs between the eight analysed studies.

**Table 4:** High-throughput studies on microRNAs.

**Author**	**Nr of patients**	**Neo**	**Adj**	**Method → validation method**	**High expression microRNAs → favorable survival**	**High expression microRNAs → unfavorable survival**
Kahlert et al. (55)	30	18	16	microarray –> qPCR	In tumour invasion front^a^: let-7	In liver invasion front ^b^: 19b and 194
Pizzini et al. (56)	46	NR	NR	microarray –> qPCR		10b
Manceau et al. (57)	132	132	NR	microarray –> qPCR		31-3p
Ellermeier et al. (58)	27	NR	NR	qPCR array	9	***125a-5p, 145, 199a-5p**,* 323-3p, ***99b***
Li et al. (59)	48	22	NR	microarray –> qPCR	***99b-5p***	
Pecqeux et al. (60)	25	NR	NR	microarray –> qPCR	In adjacent liver: ***125***, 127, ***145***, 192, 194, ***199-5***, 215, 429	
					In stroma CRLM: ***199-3***	
Kingham et al. (61)	91	53	58	NGS –> qPCR		203
Li et al. (62)	48	NR	NR	microarray –> qPCR	196b-5p	

*Eight studies analysed microRNA expression by high-throughput methods. The microRNAs showed in bold italics are differentially expressed in more than one study. Neo, neoadjuvant chemotherapy administered; Adj, adjuvant chemotherapy administered; NR, not reported*.

a *Tumour invasion front = first 10 cell rows into the tumour measured from the tumour/liver transition border*.

b *Liver invasion front = first 10 cell rows into the liver measured from the tumour/liver transition border*.

For many microRNAs, the precise mRNA target is not known. Three papers review microRNAs in the light of immune responses (63), tumour attack and immune escape (64), and PD-1/PD-L1 immune checkpoints (65), giving an overview on the interaction between microRNAs and the immune system. Although there is no overlap in the microRNAs that are differentially expressed in the studies, the downstream effect of the differentially expressed microRNAs might be similar (Supplementary Table 2).

## Discussion

We have reviewed the prognostic value of tissue-based immune-related markers after surgery for CRLM. We find that CD3, CD4, and CD8 are the most frequently studied protein markers (Supplementary Table 1). These T cell markers represent the most abundant immune cell types in the tumour microenvironment of CRLM, and are therefore logical markers to study. However, this means that certain immune cell types remain under-analysed in relation to patient survival. The data generated by analysed studies are based on diverse and heterogeneous patient populations as well as various treatment characteristics. This prohibits drawing firm conclusions on the role of the immune system in patients with colorectal liver metastases. Therefore, standardising the quantification of immune infiltration would make it easier to compare studies with each other. In primary colorectal cancer, the consensus Immunoscore is proposed to be implemented in the current TNM staging. This Immunoscore quantifies CD3+ and CD8+ T cells by digital pathology software and has prognostic value and might contribute to compare the results among various study groups (29).

### Intra-Tumoural CD3+ T Cells

High numbers of intra-tumoural CD3, CD4, and CD8 were predictive of a favourable patient survival. Out of the eight studies (11, 13, 15–17, 20, 30, 31) that combined immune markers to create the best predictor of patient survival, six include intra-tumoural CD3+ quantification (13, 15–17, 30, 31). These results suggest that intra-tumoural CD3+ T cells have the best prognostic value. However, there are differences in patient and treatment characteristics between the studies. For example, in the studies that observed prognostic value in intra-tumoural CD3+ quantification, neoadjuvant chemotherapy was more often administered compared to studies that did not observe prognostic value (74.9% vs. 31.5%). Previous studies have shown that chemotherapeutic regimens including oxaliplatin or anthracyclins can induce an immune response leading to immunogenic tumour cell death (66–69). This suggests that patients who receive these neoadjuvant treatments potentially have more immune infiltration, e.g., due to an increase in tumour antigen-presentation via MHC (70, 71). However, the majority of the studies included in our review do not explain treatment regimes in detail and do not show associations of neoadjuvant chemotherapy with immune infiltration or patient survival. Thus, the differences in treatment regimes of administering neoadjuvant chemotherapy may have caused bias.

### Univariable vs. Multivariable and Clinicopathological Characteristics

Several studies only show univariable analysis of their targeted marker without showing multivariable analysis. This makes it difficult to estimate the importance of the targeted molecular marker in the light of known clinicopathological risk factors. In the studies that do find a significant association between patient survival and an immunological marker, clinicopathological risk factors can also be predictive of patient survival or are strongly related to the immune marker. Out of the 10 studies that analyse CD3/CD4/CD8/FOXP3 by IHC, six studies show multivariable analyses including clinicopathological variables (11, 13, 15, 16, 18, 21). Out of those six studies, four show that known clinical risk factors are also independent predictors of patient survival (11, 16, 18, 21). For example, Katz et al. show by multivariable analysis that high intra-tumoural CD3 expression is associated with a favourable patient survival, but so is the clinical risk score (72). In fact, the clinical risk score is an even better predictor of patient survival compared to high intra-tumoural CD3 expression [*p* < 0.001; odds ratio 8.8 (3.3–23.5) vs. *p* = 0.04; odds ratio 4.2 (1.1–16.9)] (11).

### Other Molecular Prognostic Markers

Multiple additional molecular markers have shown prognostic value after surgical resection of colorectal liver metastases. It is known that mutations in the KRAS (codon 12 and 13) and BRAF (V600E) genes are associated with unfavourable patient survival (7). In addition, several molecular characteristics are positively associated with an enhanced immune response in (metastatic) colorectal cancer and favourable patient survival (73), like the CpG island methylator phenotype (CIMP) (74) and the microsatellite instability phenotype (75). Patients with microsatellite instable tumours typically have less often metastases, a better survival and a good response to modern immunotherapy (76). Although we primarily studied immune related markers on protein and RNA level, alterations at the DNA level, not discussed in this review, could potentially also be relevant for prognostication (7).

### Applicability as Prognostic Biomarker

Modern high-throughput methods like next generation sequencing or microarrays are innovative, but not every hospital has the equipment and knowledge to apply them in a diagnostic setting. Thus, although IHC is a relatively old technology, it is the most used method to predict patient survival. The high-throughput studies of both mRNA and miRNA in CRLM find little overlap in prioritising genes. In contrast, gene signatures designed by high-throughput methods are used to guide treatment decisions in breast cancer: this signature (MammaPrint) is based on the expression of 70 genes and is used to assess whether patients could benefit from adjuvant chemotherapy (77). One could speculate whether high-throughput studies are worthwhile, since genetic markers have the problem that there are potentially many markers. Thus, you need to build up a catalogue of all possible genetic markers, which takes time and cohorts of sufficient size. In contrast, every patient has an immune response (whether effective or not), making immune-related markers as the more obvious immediate target.

## Conclusions and Future Perspective

The most important IHC-marker for prognostication in the studies we reviewed is CD3, a general T cell marker. Although intra-tumoural CD3+ quantification is the best immune-related predictor of patient survival, we think that this marker is not accurate enough to guide individual treatment decisions. In addition, while high-throughput expression studies show promise, there is no consensus yet about the important genes for prognostication. A high CXCR4 expression was reported by several studies as an unfavourable prognostic factor, and this might be a future target for improving the efficacy of immunotherapy (37). We know that patients with immunocompetent tumours can potentially benefit from immunotherapy (78, 79). Hopefully, future therapy can enhance the function of the immune system in an effective way to eradicate cancer in all tumour-bearing organs.

## Author Contributions

JH performed the analysis and wrote the manuscript. KdJ devised the main conceptual ideas and supervised JH. KK and RS helped supervise the project. All authors discussed the results and contributed to the final manuscript.

### Conflict of Interest Statement

The authors declare that the research was conducted in the absence of any commercial or financial relationships that could be construed as a potential conflict of interest.
