# Supplementary material for: Systematic Review of the Prognostic Role of the Immune System After Surgery of Colorectal Liver Metastases
Source: Front Oncol. 2019 Mar 19;9:148. doi: 10.3389/fonc.2019.00148 (PMC6433783; doi:10.3389/fonc.2019.00148)
Supplement: Supplementary file 1 [file Table_1.docx]

| **microRNAs** | **Immune function Paladini** ^1^ | **Immune function Eichmüller** ^2^ | **Immune function Wang** ^3^ |
| --- | --- | --- | --- |
| **let-7** | Cell maintenance hematopoietic stem cells  Cell function and polarization macrophages Cell differentiation cytotoxic T-cells |  |  |
| **9** | Cell function/polarization macrophages  Cell activation B cells | Tumor immune escape by targeting genes: B2M, TAP-1, PSMB8, PSMB10, HLA-B, HLA-C, HLA-F, HLA-H in nasopharyngeal cancer |  |
| **10b** | Suppression of NK-mediated killing of tumor cells in breast cancer | Tumor immune escape by targeting MICA and MICB in various cancers | Mir-10a regulates PTEN in NSCLC |
| **19b** | Cell differentiation T helper 17 cells |  | PTEN in gastric cancer |
| **31-3p** |  |  |  |
| **99b-5p** |  |  |  |
| **99b** | Cell maintenance hematopoietic stem cells |  |  |
| **103-2** | Mir-103 🡪 Cell development granulocyte–macrophage progenitors |  |  |
| **125a-5p** |  |  |  |
| **125** | Cell maintenance hematopoietic stem cells  Cell polarization macrophages  Cell activation B cells  Cell differentiation T helper cells | Tumor immune escape by targeting IRF-4 in macrophages |  |
| **127** |  |  |  |
| **145** |  | Tumor immune escape by targeting STAT1 in colorectal cancer | Same study reference as in Eichmüller et al. |
| **196b-5p** | Cell development multipotent progenitors  Cell differentiation monocytes  Cell differentiation granulocytes |  |  |
| **192** |  |  |  |
| **194** |  |  |  |
| **199-5** |  |  |  |
| **199a-5p** |  |  |  |
| **199-3** | Cytokine production, ↑IKKβ (direct upstream activator of NF-kB pathway) |  | Mir-199a-3p regulates MTOR in endometrial cancer cells |
| **203** |  | Inhibitory effects on dendritic cell function in pancreatic cancer |  |
| **215** |  |  |  |
| **323-3p** |  | Mir-323b-5p 🡪 tumor immune escape by targeting B7/CD28 in colorectal cancer |  |
| **429** |  |  |  |

**Supplementary table 1. MicroRNAs and their proposed immunological function.**

In the left column, microRNAs found to be differentially expressed (table 4) regarding patient survival are summarized, with their proposed immunological function according to three recent review articles.

**Bibliography**

1. Paladini L, Fabris L, Bottai G, Raschioni C, Calin GA, Santarpia L. Targeting microRNAs as key modulators of tumor immune response. J. Exp. Clin. Cancer Res. 2016;35(1):103. doi:10.1186/s13046-016-0375-2

2. Eichmüller SB, Osen W, Mandelboim O, Seliger B. Immune Modulatory microRNAs Involved in Tumor Attack and Tumor Immune Escape. JNCI J. Natl. Cancer Inst. 2017;109(10). doi:10.1093/jnci/djx034

3. Wang Q, Lin W, Tang X, Li S, Guo L, Lin Y, Kwok H. The Roles of microRNAs in Regulating the Expression of PD-1/PD-L1 Immune Checkpoint. Int. J. Mol. Sci. 2017;18(12):2540. doi:10.3390/ijms18122540
